# Modulation of cerebral cortex activity by acupuncture in patients with prolonged disorder of consciousness: An fNIRS study

**DOI:** 10.3389/fnins.2022.1043133

**Published:** 2022-11-29

**Authors:** Yiwei Liu, Ning Sun, Jing Xiong, Yuanfang Zhou, Xiangyin Ye, Hua Jiang, Hua Guo, Na Zhi, Jingkang Lu, Peijue He, Huilin Yang, Qingbin Li, Ruirui Sun, Jing He

**Affiliations:** ^1^Rehabilitation Medicine Center and Institute of Rehabilitation Medicine, West China Hospital, Sichuan University, Chengdu, Sichuan, China; ^2^Key Laboratory of Rehabilitation Medicine in Sichuan Province, Chengdu, Sichuan, China; ^3^Acupuncture and Tuina School/The 3rd Teaching Hospital, Chengdu University of Traditional Chinese Medicine, Chengdu, Sichuan, China

**Keywords:** acupuncture, neuroplasticity, functional near-infrared spectroscopy (fNIRS), prolonged disorder of consciousness, dorsolateral prefrontal cortex (DLPFC)

## Abstract

**Background and objective:**

Acupuncture is a promising non-pharmacological therapy for patients with prolonged disorder of consciousness (PDOC); however, its underlying mechanism remains uncertain. This study aimed to reveal the modulatory effects of acupuncture on the cerebral cortex activity among patients with PDOC.

**Materials and methods:**

Twenty-eight PDOC patients were randomly assigned to the treatment (*n* = 14) or control (*n* = 14) group. The treatment group received one session of acupuncture, while the control group received one session of sham acupuncture. All patients underwent evaluation of the functional connectivity and activation response of the dorsolateral prefrontal cortex (DLPFC), primary motor cortex (M1), and primary somatosensory cortex (S1) *via* functional near-infrared spectroscopy. We further explored the potential correlation of the consciousness level and activation response/functional connectivity with acupuncture.

**Results:**

Compared to the control group, a single session of acupuncture significantly tended to enhance resting-state functional connectivity (rsFC) in DLPFC-M1, DLPFC-M1, and S1-S1. And the activation level of the DLPFC (both sides) in the acupuncture group is significantly higher than those in sham acupuncture group. However, no significant correlation was found between the consciousness level and activation response/functional connectivity.

**Conclusion:**

One session of acupuncture has a significant modulation of rsFC and activation in the DLPFC, M1, and S1 with PDOC patients. Acupuncture-evoked effect may have some functional significance in PDOC patients. This is an important step toward exploring the acupuncture effects on PDOC patients.

## Introduction

Disorder of consciousness (DOC) refers to an altered state of consciousness usually caused by damage or dysfunction of the brain region that regulates arousal and consciousness (Schiff and Plum, 2000; Giacino et al., 2014). Although many patients have little or no long-term sequelae, some suffer from a prolonged DOC (PDOC), such as a vegetative state/unresponsive wakefulness syndrome (VS/UWS) or minimally conscious state (MCS). VS/UWS refers to a condition of wakefulness without awareness. MCS patients show unequivocal signs of non-reflex cortically mediated behaviors. The prevalence of vegetative state and unresponsive wakefulness syndrome is estimated 0.1–0.2 cases per 100,000 members (van Erp et al., 2014, 2015). The main goal of rehabilitation for patients with PDOC is to improve their level of alertness.

In addition to multiple drug and electrophysiological interventions (Thibaut et al., 2019), non-drug therapies, such as acupuncture (Zhang et al., 2019), have also been used to treat patients with PDOC. Acupuncture is a traditional Chinese medical treatment that involves the insertion of fine needles into certain body parts. Recently, many studies have reported that acupuncture can improve the symptoms of patients with neurological or psychiatric disorders, including sensory and cognitive functions (Jia et al., 2017; Ormsby et al., 2020; Huang et al., 2021; Mao et al., 2021). Although only few studies have evaluated the role of acupuncture in PDOC patients, they have shown encouraging effects. A study reported that amantadine combined with acupuncture restored the consciousness more rapidly than amantadine alone during the first 4 weeks of treatment (Yang et al., 2020).

Acupuncture increases the cerebral blood flow and cerebral glucose metabolism by modulating the sympathetic nervous system (Li et al., 2013; Benrick et al., 2017). Neiguan (PC6) is the Luo-connecting point of the Pericardium meridian used to treat consciousness for decades. A functional magnetic resonance imaging study confirmed that acupuncture of PC6 activated multiple brain regions (Yoo et al., 2004). For example, acupuncture of PC6 activated the temporal lobe, frontal lobe, cingulate gyrus, and cerebellum of Alzheimer’s disease patients to varying degrees (Fu et al., 2005). Acupuncture of PC6 significantly improves the brain pathology of the injured brain areas and stimulates the neurological function recovery (Zhou et al., 2010). To study the neuromodulation effects of acupuncture, it is necessary to investigate its underlying mechanism, including its effects on brain communication and neuronal activity in PDOC patients.

Functional near-infrared spectroscopy (fNIRS) is a non-invasive optical neuroimaging technology, which is portable, cheap, and wearable and has few contraindications. It can be continuously and repeatably performed in natural and clinical environments (Gratton et al., 1995; Villringer and Chance, 1997; Strangman et al., 2002). In addition, fNIRS can assess the brain function in DOC patients (Kempny et al., 2016; Zhang et al., 2017) and is suitable for the evaluation of regional neural dynamics related to the entire acupuncture operation process (i.e., needle insertion, rotation, and retrieval) (Wong et al., 2021; Zhang T. et al., 2021). It is indispensable for the evaluation of the real-time efficacy of acupuncture in PDOC patients.

The present study is the first to investigate the regulatory effect of acupuncture on the cerebral cortex of PDOC patients using fNIRS. In addition, cortical activity and functional connectivity may be related to the severity of the abnormality of consciousness level. Consequently, we studied the underlying relationship between the consciousness level and acupuncture modulation response/functional connectivity.

## Materials and methods

### Participants

A total of 28 consecutive patients who met the criteria for PDOC by the European Academy of Neurology guidelines (Kondziella et al., 2020) were enrolled from the department of rehabilitation of West China Hospital between February 2021 and May 2022. Of the 28 patients, 14 underwent acupuncture treatment (AT) and 14 underwent sham acupuncture treatment (SA). No significant differences were found between the AT and SA groups in terms of their age, sex, disease duration, baseline Coma Recovery Scale-Revised (CRS-R), or Glasgow Coma Scale (GCS) scores (Table 1). The study protocol was reviewed and approved by the Ethics Committee Biomedical Research, West China Hospital of Sichuan University (approval no. 20201070). The study was registered at the Chinese Clinical Trial Registry (registration no.: ChiCTR2100053355).

**TABLE 1:** Baseline characteristics.

Parameters	AT group (*n* = 14)	SA group (*n* = 14)	*P*-value
Age (years)	62.0 ± 15.6	48.9 ± 20.2	0.103
Gender (M/F)	10/4	8/6	0.127
Duration of disease (days)	96.4 ± 113.5	85.7 ± 82.2	0.825
**Etiology**			
Traumatic injury	3 (21%)	4 (29%)	0.663
Hemorrhagic injury	6 (43%)	4 (29%)	0.430
Ischemic injury	5 (36%)	6 (42%)	0.699
**Clinical assessment**			
COMA	4 (29%)	5 (37%)	0.686
VS/UWS	4 (29%)	3 (21%)	0.663
MCS	6 (42%)	6 (42%)	1.000
Baseline CRS-R total score	5.6 ± 3.7	7.6 ± 4.9	0.340
Baseline GCS total score	6.8 ± 2.3	7.7 ± 2.7	0.213

Duration: time at which the patient has been in a disorder of consciousness. AT, acupuncture group; SA, sham acupuncture group; F, female; M, male; VS, vegetative state; UWS, unresponsive wakefulness syndrome; MCS, minimally conscious state; CRS-R, the coma recovery scale-revised; GCS, the Glasgow coma scale.

### Inclusion criteria

Patients were included if they: (1) were aged 18–75 years, (2) right-handed, (3) met the criteria of PDOC (>4 weeks since brain injury), (4) had a clear etiology and stable vital signs, (5) had not participated in other clinical investigations within the prior 3 months, and (6) signed an informed consent form by a family member or authorized person.

### Exclusion criteria

Patients were excluded if they: (1) with unstable condition, serious and uncontrolled complications of the respiratory or circulatory system, acute phase of disease onset, status epilepticus, polytrauma, limb fracture, large skin defect; (2) with mental illness, drug or long-term alcohol addiction, severe endocrine or metabolic diseases, space-occupying lesions that may affect the study results; (3) with intracranial tumor, intracranial infections; (4) pregnancy; and (5) with cranial defects or abnormalities, unable to collect NIRS data.

### Intervention

**FIGURE 1:**
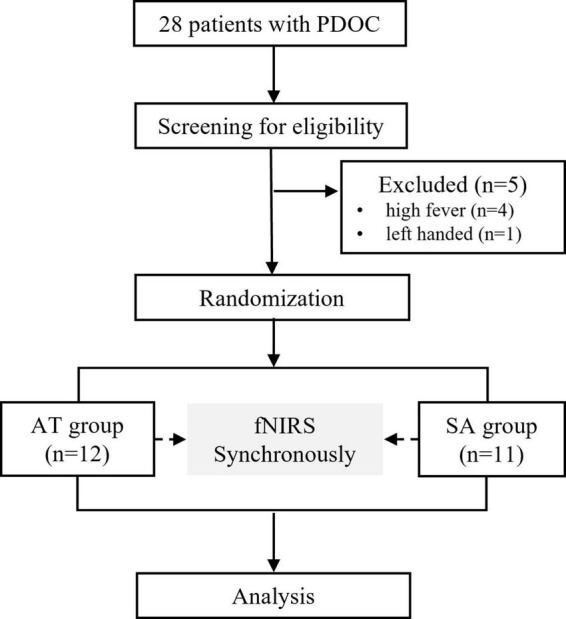
CONSORT flow diagram. PDOC, prolonged disorder of consciousness; fNIRS, functional near-infrared spectroscopy; AT, acupuncture treatment; SA, sham acupuncture.

The participants were randomly allocated to the AT or SA groups using a random number table. The assessors were blinded throughout the whole study. Before acupuncture, patients underwent a 1-week screening, which included behavioral assessment and routine laboratory studies, to assess their stability. After the screening, we excluded five patients due to high-grade fever and left-handedness (Figure 1). All patients were continued on their original medication regimen. The trial was conducted in accordance with the Declaration of Helsinki.

**FIGURE 2:**
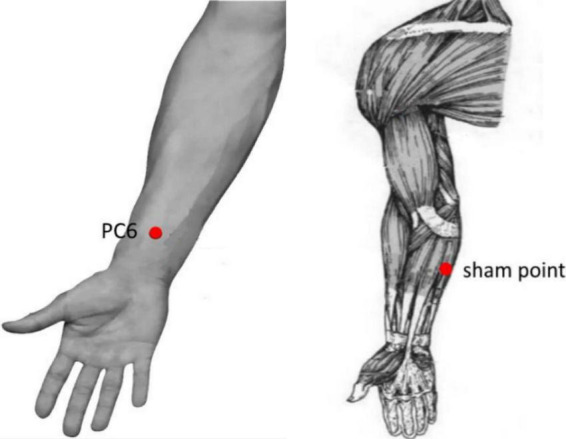
Location of acupoint or sham point. The location of Neiguan (PC6) acupoint is 2 cm above the transverse crease of the wrist, between the tendons of m. palmaris longus and m. flexor carpi radials. The sham point is on the ulnar side of the arm, midway between the epicondylus medialis of the humerus and the ulnar side of the wrist.

**FIGURE 3:**
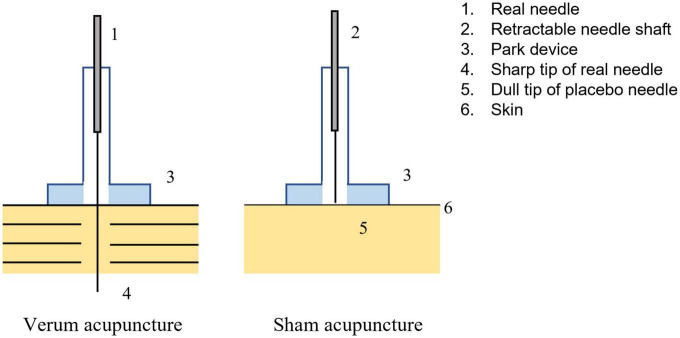
Verum acupuncture and Sham acupuncture.

Participants received one session of acupuncture either on the right PC6 or on the sham point, which was performed by trained, licensed acupuncturists with at least 3 years of clinical experience in acupuncture. The location of PC6 is 2 cm above the transverse crease of the wrist, between the tendons of m. palmaris longus and m. flexor carpi radials. The sham point is on the ulnar side of the arm, midway between the epicondylus medialis of the humerus and the ulnar side of the wrist (Zhao et al., 2019; Figure 2). The SA group received non-inserted acupuncture using the Park sham needle supported by the Park device (Park et al., 2002; Figure 3).

### Acupuncture administration

**FIGURE 4:**
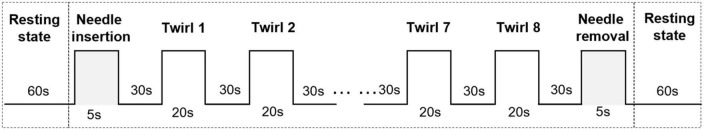
Experimental paradigm. The first task was needle insertion (NI). The second task was needle twirl, which was repeated eight times (T1, T2……T8). The last task was needle removal (NR).

Both groups underwent similar acupuncture procedure and manipulation. For the acupuncture procedure, three different acupuncture manipulations were used. Specifically, the first manipulation was needle insertion (NI), followed by an initial twirl to activate the acupoint. The second manipulation was a needle twirl, which was repeated eight times (T1, T2……T8) to keep the acupoint activated. The duration of the second task was 400 s, including four twirls (8 × 20 s) and three rest times (8 × 30 s). Finally, the third manipulation was needle removal (NR). The acupuncture procedure lasted for a total of 8 min and 50 s (Figure 4). The acupuncture procedure used in the present study was in line with previous fNIRS studies (Fernandez Rojas et al., 2019; Zhang T. et al., 2021).

Disposable sterile filiform needles (0.25 × 40 mm; Jiajian, Wuxi, China) with Park devices were used for the AT group and inserted perpendicularly into the point at a depth of 16–33 mm after skin disinfection using alcohol. During each needle twirl, the needle was bi-directionally twisted within 90°–180°, lifted, and thrust at an amplitude of 3–5 mm for 60–90 times/min to induce and maintain the *deqi* sensation (including soreness, numbness, distention, and heaviness).

### Safety and behavior assessment

For baseline assessment, two blinded assessors independently performed the CRS-R and GCS assessments in a randomized order, thus permitting interrater comparisons. The CRS-R consists of 23 hierarchically arranged items that comprise six subscales that evaluate the auditory, visual, motor, verbal, communication, and arousal functions (Giacino et al., 2004). The GCS is scored between 3 and 15, with 3 being the worst and 15 the best. It is composed of three parameters: best eye, best verbal, and best motor responses (Bodien et al., 2021). After acupuncture, the staff completed a safety assessment.

### Functional near-infrared spectroscopy imaging and data collection

In the experiment, a 20-channel fNIRS system (OMM-3000; Shimadzu, Kyoto, Japan) with continuous-wave laser diodes of 780, 805, and 830 nm wavelengths was used. Eight emitters and seven detectors were used. The optoelectronic device was placed on top of the international 10–20 electroencephalogram (EEG) electrode placement system using a separate sized cap selected based on head circumference (Okamoto et al., 2004). For accurate fixation, the photoelectric device was installed on the bracket inside the NIRS cap, and the distance between the light source and photoelectric device of the detector was set at 2.5–3 cm (Gratton et al., 2006). The sampling rate was 13.33 Hz.

**FIGURE 5:**
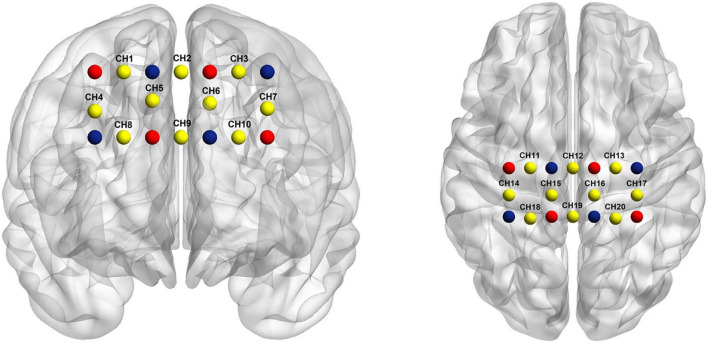
Optode probe set. The dorsolateral prefrontal cortex (DLPFC) (channels 1–7), primary motor cortex (channels 10–17), and primary somatosensory cortex (channels 18–20). Channels (in yellow) are measured between emitters (in red) and detectors (in blue).

We defined the dorsolateral prefrontal cortex (DLPFC, channels 1–7), primary motor cortex (M1, channels 10–17), and primary somatosensory cortex (S1, channels 18–20) as regions of interest (Figure 5). The transmitter position used Fpz and Cz as reference points. DLPFC was selected because it is involved in the cortico-subcortical network and has strong connections to the thalamus and striatum, which are impaired in DOC based on the mesocircuit fronto-parietal model (Giacino et al., 2014). Moreover, it plays a critical role in executive functioning and higher-order mental activities (Liu et al., 2018; Matyi et al., 2021). During acupuncture, fNIRS simultaneously measured the brain activity. There were 60 s of resting-fNIRS collected before and after AT.

### Functional near-infrared spectroscopy data analysis

The near-infrared spectral signal was quantified using HOMER2 and NIRS-SPM v4.1 toolboxes to assess the activation map of oxy-hemoglobin (oxy-Hb) levels. Previous research used oxy-Hb instead of deoxy-hemoglobin (deoxy-Hb) as oxy-Hb signal has a better signal-to-noise ratio than deoxy-Hb signal (Sato et al., 2004) and is considered the most sensitive indicator of regional cerebral blood flow (Hoshi et al., 2001).

For pre-processing, we transformed the original NIRS light intensity into an optical density signal. For filtering, the channels containing undesirable signals and prolonged motion artifacts were identified, deleted, and recalibrated; then, the HOMER2 built-in functions (parameters set as tMotion = 1.0; tMask = 1.0) and a bandpass filter were used to reduce the physiological artifacts (0.01–0.1 Hz cut-off frequency). The data from each channel were visually inspected to exclude the channels with excessive noise from subsequent analysis. The modified Beer–Lambert law was adopted to transform the filtered optical density data into the oxy-Hb concentration (Delpy et al., 1988). The time window before and after the peak of hemodynamic response (2–15 s after the onset of acupuncture stimulation) was selected for statistical analyses.

The hemodynamic changes after acupuncture were transformed into coherence coefficients to represent the functional connectivity strength among the 20 channels. In addition, the general linear model (GLM) method was used to analyze the fNIRS time series (Plichta et al., 2007). The experimental design matrix for GLM analysis included three predictors representing three different acupuncture manipulations (NI, needle twist, and NR). Needle twirl-related β-values were one of the weights of GLM, which represented the change in oxy-Hb level at the inflection point correlation stage and was calculated for each channel. Needle twirl was a critical factor to induce and maintain the *deqi* sensation. Therefore, we used needle twirl-related β-values as the study data.

Meanwhile, the rotation-related β value of each channel was analyzed using one-sample *t*-test and the false discovery rate (FDR) method (Benjamini and Hochberg, 1995) (*p* < 0.05). Independent samples *t*-test was used to compare twirl-related β-values between AT and SA groups. Pearson’s correlation was used to study the relationship between CRS-R/GCS baseline score and twirl-related β-values. The significance level was set at *p* < 0.05.

## Results

Among the 23 eligible PDOC patients, 12 from the AT group and 11 from the SA group underwent fNIRS. Of the 23 records, 5 raw data (four from the AT group and two from the SA group) were excluded from analysis because of low signal quality due to excessive head movements. Thus, data were available for a total of 17 PDOC patients (eight AT patients and nine SA patients).

### Resting-state functional connectivity

The changes in resting-state functional connectivity (rsFC) were assessed immediately after acupuncture. The hemodynamic changes after acupuncture were transformed into coherence coefficients. The rsFC was compared between the two groups from the perspective of consistency.

**FIGURE 6:**
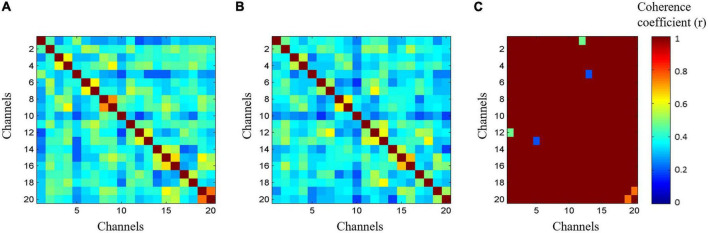
The resting-state functional connectivity (rsFC) in two groups. Data were extracted from 60 s functional near-infrared spectroscopy (fNIRS) oxy-hemoglobin results after acupuncture. **(A)** Coherence matrices of acupuncture treatment group. **(B)** Coherence matrices of sham-acupuncture treatment group. **(C)** Coherence matrices within the groups.

In the AT and SA groups, the oxy-Hb coherence matrices were mapped within the groups (Figure 6). The rsFC of DLPFC-M1 (1, 12), DLPFC-M1 (5, 11), and S1-S1 (19, 20) channel pairs in the AT group was significantly higher than that in the SA group (*r* = 0.5–0.8, FDR corrected *p* < 0.05).

### Cortical activation

**FIGURE 7:**
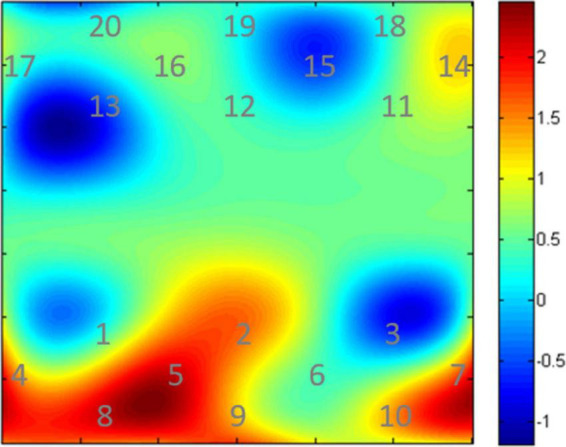
Cortical activation maps of oxy-Hb level. The digits shown in represent the channel numbers. The bar graph (in the right side) shows the *t* values.

Our results showed that twirl-related β-values in DLPFC (channels 5 and 7) were higher in the AT group than in the SA group, and the difference was statistically significant (*p* < 0.05 after FDR correction). The activation diagram of oxy-Hb level by paired *t*-tests during acupuncture is shown in Figure 7.

### Correlations between coma recovery scale-revised/Glasgow coma scale score and functional near-infrared spectroscopy data

There was no correlation between the fNIRS data (rsFC and cortical activation) in the AT group and baseline CRS-R/GCS scores in PDOC patients (FDR corrected *p* > 0.05).

## Discussion

A single acupuncture session significantly increased the activation of the DLPFC region. The rsFC in the DLPFC, MA, and S1 was significantly enhanced after acupuncture. However, no association was found between fNIRS data and baseline CRS-R/GCS scores. These results suggest that acupuncture can cause hemodynamic activation of the DLPFC region and regulate the connectivity between DLPFC, MA, and S1. Therefore, acupuncture may have certain functional significance in patients with PDOC.

Previous studies reported that the cerebral cortical activity was decreased in the posterior and anterior cingulate cortex, prefrontal cortex, and angular gyrus in PDOC patients (Hannawi et al., 2015). Meanwhile, in PDOC patients, regions closely related to the fronto-parietal control networks (FPCNs) were damaged (Crone et al., 2013). The involvement of multifunctional networks may be related to the clinical features of PDOC (Mencarelli et al., 2020). We selected our regions of interest (M1, S1, and DLPFC) based on full consideration and literature review. S1 plays a critical role in conscious tactile perception (Kulkarni et al., 2005). M1 promotes motor and cognitive function (Sanes and Donoghue, 2000). DLPFC provides the central integrated function of motor control and behavior and is an important component of the decision network (Heekeren et al., 2006). The right DLPFC has been linked to maintenance of sustained arousal and attention (Sturm and Willmes, 2001), which is similarly relevant for PDOC patients. We speculated that the modulation in rsFC and cortical activation was owing to the rebalancing effects of acupuncture.

The significant hemodynamic activation of DLPFC and rsFC in DLPFC, MA, and S1 found in our study may suggest a mechanism for acupuncture treatment of PDOC. M1, S1, and DLPFC are commonly used neuroanatomical sites for electrophysiological intervention in PDOC, and some studies have confirmed the effectiveness of stimulating these sites (Cincotta et al., 2015; Naro et al., 2015; Xia et al., 2017; Ro and Koenig, 2021). In our research, rsFC and cortical activation were noted in response to PC6 neuromodulation and acupuncture stimulation, despite the lack of precise stimulation. The phenomenon may be explained by functional correlations due to cascades of several intermediates or relay processes transmitted *via* the cortical-subcortical cycle (Honey et al., 2009). For example, PDOC has also been suggested to be associated with increased temporal activity during passive tasks and decreased relationships between internal networks (subcortical areas), which may imply reduce functional relationships between these structures (Mencarelli et al., 2020). Meanwhile, we speculated that the modulation in rsFC and cortical activation was owing to the rebalancing effects of acupuncture because the therapeutic benefits of AT and its purported clinical efficacy suggests that acupuncture acts to maintain a homeostatic balance of the internal state (Ghafoor et al., 2019; Zhang Y. et al., 2021).

This study has certain limitations. First, limited optical channels were used. Only DLPFC, S1, and M1 were evaluated. In future studies, more brain regions should be assessed using advanced fNIRS systems. Second, the time range recorded by fNIRS was not uniform. Although set and programmed, hemoglobin concentrations can vary between different times of the day. The time range recorded by fNIRS needs to be consistent in further studies. Third, we selected a single acupuncture point to evaluate the regulatory effect of acupuncture on the cerebral cortex, without observing the clinical efficacy in PDOC patients. Future studies should include observations of clinical efficacy. Finally, the sample size was small. Therefore, these results should be considered preliminary. Subsequent studies should have a larger sample size and should replicate the study in patients with PDOC to determine the efficacy and possible mechanisms of acupuncture.

To the best of our knowledge, our study is the first to use fNIRS to evaluate the ability of acupuncture to evoke cortical activity and functional connectivity in PDOC patients. This fNIRS study is an important step toward exploring the acupuncture effects on PDOC patients. To clarify the effects of acupuncture further and develop its suitable clinical applications, we aim to develop advanced techniques to assess the functional signals and study the acupuncture mechanism in PDOC patients.

## Conclusion

We found that in PDOC patients, a single acupuncture session has a regulatory effect on hemodynamic activation and rsFC in DLPFC, MA, and S1 by using fNIRS. Our results may suggest that the ability of acupuncture to evoke cortical activity and functional connectivity in PDOC patients. However, the neuromodulation effects of acupuncture on PDOC patients need to be studied further.

## Data availability statement

The raw data supporting the conclusions of this article will be made available by the authors, without undue reservation.

## Ethics statement

The studies involving human participants were reviewed and approved by the Ethics Committee Biomedical Research, West China Hospital of Sichuan University (approval no. 20201070). The patients/participants provided their written informed consent to participate in this study.

## Author contributions

All authors listed have made a substantial, direct, and intellectual contribution to the work, and approved it for publication.

## Abbreviations

DOC: disorder of consciousness
PDOC: prolonged DOC
PC6: Neiguan
fNIRS: functional near-infrared spectroscopy
AT: acupuncture treatment
SA: sham acupuncture treatment
CRS-R: Coma Recovery Scale-Revised score
GCS: Glasgow Coma Scale score
NI: needle insertion
NR: needle removal
DLPFC: the dorsolateral prefrontal cortex
M1: the primary motor cortex
S1: the primary somatosensory cortex
oxy-Hb: oxy-hemoglobin
deoxy-Hb: deoxy-hemoglobin
GLM: general linear model
FDR: false discovery rate
rsFC: resting-state functional connectivity
FPCNs: the fronto-parietal control networks.
